# Effect of decreased platelets on postoperative recurrence of chronic subdural hematoma

**DOI:** 10.3389/fneur.2023.1308991

**Published:** 2023-12-20

**Authors:** Kenji Yagi, Maoki Matsubara, Eiichiro Kanda, Yukari Minami, Tomohito Hishikawa

**Affiliations:** ^1^Department of Neurosurgery, Kawasaki Medical School, Kurashiki, Okayama, Japan; ^2^Department of Medical Science, Kawasaki Medical School, Kurashiki, Okayama, Japan

**Keywords:** chronic subdural hematoma, postoperative recurrence, eosinophil, platelet, burr hole surgery

## Abstract

**Introduction:**

Chronic subdural hematoma (CSDH) is commonly treated using simple burr hole surgery. However, postoperative recurrence occurs at a relatively high rate of 10–20%. A decrease in platelet count (PC) may be associated with recurrence via a hemostasis disorder; however, this association has not been well-studied. Therefore, this study aimed to investigate the association between PC and postoperative CSDH recurrence.

**Methods:**

We retrospectively reviewed the data for CSDHs in 488 cerebral hemispheres of 431 patients who underwent burr hole surgery at our institution between January 2013 and December 2022. The association between preoperative PC and postoperative CSDH recurrence was investigated. We used the first quartile of PC, PC < 170 × 10^3^/μL to define a threshold for decreased PC.

**Results:**

In total, 459 cerebral hemispheres with CSDHs in 405 patients were followed up postoperatively for at least 3 months or until CSDH disappeared. CSDH recurred in 39 (8.5%) cerebral hemispheres. The recurrence rate was gradually increased in parallel with a decreasing PC. Among 109 CSDHs with a decreased PC (<170 × 10^3^/μL), 15 (13.8%) recurred, whereas only 24 (6.9%) of 350 CSDHs without a decreased PC recurred (*p* = 0.03). In univariable logistic analysis, eosinophil-rich blood (≥100/μL eosinophils in peripheral blood) and a decreased PC were significant risk factors. Multivariable analysis showed that eosinophil-rich blood (adjusted odds ratio, 2.51; 95% confidence interval, 1.26–4.99; *p* = 0.009) and a decreased PC (adjusted odds ratio, 2.15; 95% confidence interval, 1.07–4.35; *p* = 0.03) were independent risk factors for recurrence.

**Conclusion:**

Our study showed that a decrease in PC was associated with postoperative CSDH recurrence. Patients with CSDH and a decreased PC require careful postoperative follow-up.

## 1 Introduction

Chronic subdural hematoma (CSDH) is a common disease that is being treated surgically more frequently worldwide (1). For symptomatic CSDH, a simple surgery such as burr hole irrigation or drainage of CSDH is widely performed as a standard treatment. However, postoperative recurrence occurs at a relatively high rate of 10–20%; subsequently, these patients require additional surgery (2).

The progression and recurrence of CSDH have recently been considered to be associated with recurrent hemorrhage, fibrinolysis, inflammation, and angiogenesis (3, 4), Various risk factors have been reported, which include old age, male sex, diabetes mellitus, anticoagulant therapy, eosinophil-rich blood, and blood type A (5–7). However, the risk factors for postoperative recurrence of CSDH have not been fully established.

Platelets play a pivotal role in hemostasis, and a decrease in platelet count (PC) leads to a bleeding tendency (8). The hemostasis disorder may facilitate the progression and recurrence of CSDH via recurrent hemorrhage. However, the effect of PCs on postoperative CSDH recurrence has not been well-studied and remains unclear. Moreover, the number of platelets required to suppress postoperative recurrence is unknown.

This retrospective, exploratory study investigated the association between a decreased PC and postoperative CSDH recurrence.

## 2 Materials and methods

### 2.1 Study design

**Figure 1:**
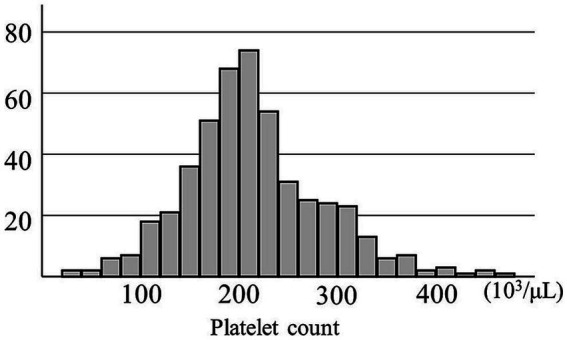
Distribution of preoperative platelet counts in 477 chronic subdural hematomas.

This study aimed to evaluate the effect of a decreased PC on postoperative recurrence of traumatic or spontaneous CSDH after the first burr hole surgery. Thus, we reviewed 488 cerebral hemispheres with CSDHs in 431 patients who were treated with burr hole surgery at our institution between January 2013 and December 2022. CSDH was diagnosed using computed tomography or magnetic resonance imaging. Four CSDHs in four patients who underwent middle meningeal artery embolization in combination with burr hole surgery were excluded. Peripheral blood was obtained at admission for examination, including preoperative platelet and eosinophil counts {median [interquartile range (IQR)], 0 day (0–1 day)}. The distribution of PCs is shown in Figure 1. Its median [IQR] was 208 [172–250] (10^3^/μL) and the mean ± standard deviation was 214 ± 70 × 10^3^/μL. However, these preoperative blood examinations were not performed in the seven CSDH surgeries in six patients; thus, they were excluded from the study. The remaining 477 CSDHs in 421 patients were included in the study (Supplementary Figure S1).

### 2.2 Definition of clinical characteristics

Hypertension was defined as systolic blood pressure of ≥140 mmHg and/or diastolic blood pressure of ≥90 mmHg or the current use of antihypertensive drugs. Diabetes mellitus was defined as a glycosylated hemoglobin A1C level of >6.5% or treatment with hypoglycemic medications. The use of antiplatelet or anticoagulant drugs was recorded. An activated partial thromboplastin time of ≥40 s or a prothrombin time with an international normalized ratio of >1.4 was defined as coagulopathy. An eosinophil count of ≥100/μL in the peripheral blood was defined as “eosinophil-rich” (7). A PC of <100 × 10^3^/μL was defined as thrombocytopenia, whereas PC of <170 × 10^3^/μL was defined as a “decreased PC,” based on the first quartile value of PCs. The preoperative CSDH volume was calculated according to the XYZ/2 method (7, 9). When the CSDHs on both sides were operated on simultaneously or the CSDH in one hemisphere was operated on within 7 days before or after surgery on the other side, the CSDH in each hemisphere was recorded as having contralateral surgery. The baseline and clinical characteristics of patients are presented in Table 1.

**Table 1:** Baseline and radiographical characteristics of 477 chronic subdural hematomas in 421 patients.

	Total (*n* = 477)
Mean age, years	78.8 ± 9.3
Male sex	327 (68.6%)
Hypertension	201 (42.1%)
Diabetes mellitus	112 (23.5%)
Use of antiplatelet drugs	67 (14.0%)
Use of anticoagulant drugs	65 (13.6%)
Coagulopathy	43 (9.0%)
Eosinophil-rich	197 (41.3%)
Platelet transfusion	5 (1.0%)
Hematoma volume (mL)	124.2 ± 42.4
Contralateral surgery	86 (18.0%)

Data are presented as the mean ± standard deviation or number (%).

### 2.3 Surgical procedure and perioperative management

Surgery with burr hole irrigation was performed under local anesthesia. CSDH was evacuated and the hematoma cavity irrigated with normal saline or artificial cerebrospinal fluid (Artcereb irrigation and perfusion solution for cerebrospinal surgery, Otsuka Pharmaceutical Factory Inc.) (10). A drainage tube was typically placed in the hematoma cavity and removed within 2 days after surgery. However, in three CSDH cases (0.6%), a drainage tube could not be placed because of the narrowing of the hematoma cavity after the evacuation of the CSDH. Because of the severely decreased PC, platelet transfusion was performed before four surgeries for five CSDHs, according to the decision of the attending physicians. To avoid postoperative thromboembolic complications (11, 12), antithrombotic therapy at presentation was continued or discontinued for only few days postoperatively (it was reinitiated soon after confirming no postoperative acute bleeding).

### 2.4 Postoperative CSDH recurrence and acute bleeding

Postoperative recurrence of CSDH was defined as symptomatic (causing severe headache, dementia, impaired consciousness, or neurological deficits such as gait disturbance or weakness in the extremities) ipsilateral enlargement of the CSDH, indicating the need for repeated surgery between 7 days and 3 months postoperatively. Postoperative early subdural hemorrhage before postoperative day 6 was considered a surgical complication but not a recurrence. On one hemisphere with CSDH, an acute subdural hematoma was found the day after the burr hole surgery; it was completely evacuated and removed via the same burr hole. Because the hematoma was not associated with a bleeding tendency, such as thrombocytopenia in PC, use of antithrombotic drugs, or coagulopathy, this acute hemorrhage was not considered as a postoperative CSDH recurrence. Patients with no recurrence were followed up postoperatively for at least 3 months or until resolution, as indicated by the disappearance of CSDH on computed tomography. Postoperative recurrence within 3 months was recorded in each hemisphere with CSDH.

### 2.5 Statistical analyses

Statistical analyses were conducted using SPSS version 28 (IBM Corp., Tokyo, Japan). Categorical variables are expressed as numbers (percentages) and numerical data as mean ± standard deviation or median (IQR). Fisher’s exact test and Student’s *t*-test were performed for intergroup comparisons. A receiver operating characteristic (ROC) curve was created from the PC for postoperative CSDH recurrence, and area under the curve and 95% confidence interval (CI) were analyzed. Logistic regression analysis was performed to investigate the risk factors for CSDH recurrence. Odds ratios (OR) were calculated using univariable and multivariable models. Variables with a *p* value of <0.10 in univariable analyses were applied for multivariable analysis. A decrease in PC was assessed as a continuous variable and categorical variable in models 1 and 2, respectively. Statistical significance was set at a *p* value of <0.05.

## 3 Results

### 3.1 Postoperative recurrence

In total, 459 cerebral hemispheres with CSDHs in 405 patients were followed up. CSDHs recurred in 39 (8.5%) cerebral hemispheres; 18 CSDHs in 16 patients were not followed up after surgery and were not included in the following analyses (Supplementary Figure S1).

### 3.2 Association of PCs and postoperative recurrence

**Figure 2:**
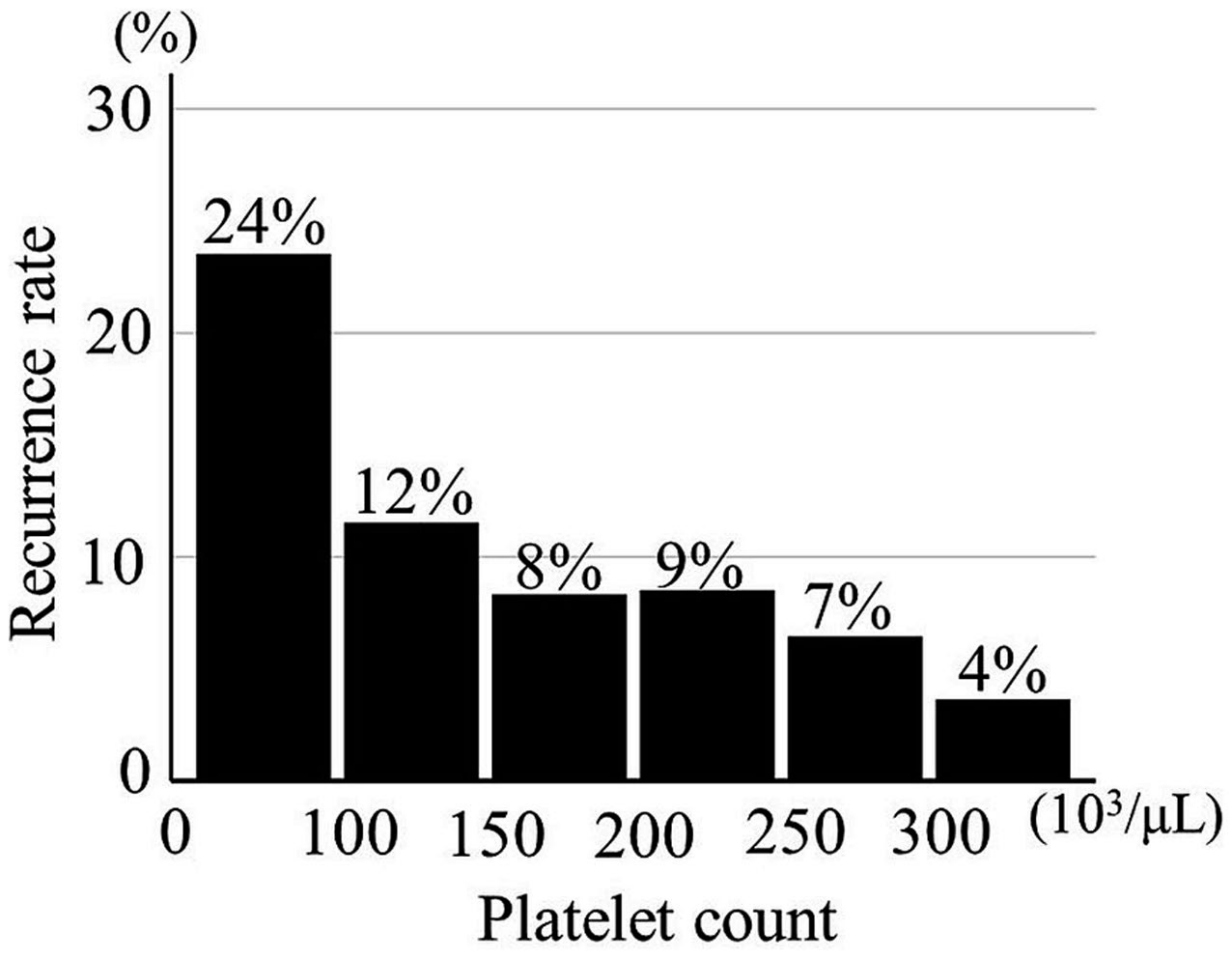
Relationship between recurrence of chronic subdural hematoma and platelet count (*n* = 459).

Platelet count was significantly lower in 39 CSDHs with recurrence than in 420 CDSHs without recurrence (189 ± 69 × 10^3^/μL vs. 216 ± 69 × 10^3^/μL, *p* = 0.02). The recurrence rate was gradually increased in parallel with a decrease in the PC (Figure 2).

An ROC curve of PCs for the CSDH recurrence was created as shown in Supplementary Figure S2. According to the distribution of PC, its first quartile was 170 × 10^3^/μL, based on which the threshold for the decreased PC (<170 × 10^3^/μL) was defined. Thereafter, the value of decreased PC was confirmed to predict the CSDH recurrence well by ROC analysis. Of 109 CSDHs with a decreased PC, 15 (13.8%) recurred; only 24 (6.9%) of 350 CSDHs without a decreased PC recurred (*p* = 0.03). The sensitivity and specificity were 38.5 and 77.6%.

### 3.3 Logistic analyses for the CSDH recurrence

Logistic analyses of postoperative recurrence were performed in 459 cerebral hemispheres with CSDHs (Table 2). In the univariable model, a decrease in the PC significantly increased the risk of recurrence; a decreased PC (<170 × 10^3^/μL) was a significant risk factor. In addition, eosinophil-rich blood was associated with the CSDH recurrence. Next, multivariable analysis was performed with the variables with a value of *p* < 0.10 in the univariable analysis. When PC was assessed as a continuous variable in model 1, a decrease in PC was associated with CSDH recurrence in model 1 (per 5 × 10^3^/μL decrease: OR, 1.36; 95% CI, 1.05–1.76; *p* = 0.02) When PC was assessed as a categorical variable in model 2, decreased PC was an independent risk factor for the recurrence (adjusted OR, 2.15; 95% CI, 1.07–4.35; *p* = 0.03). In addition, eosinophil-rich blood was an independent risk factor in both model 1 (adjusted OR, 2.63; 95% CI, 1.31–5.27, *p* = 0.007) and model 2 (adjusted OR, 2.51; 95% CI, 1.26–4.99, *p* = 0.009).

**Table 2:** Univariable and multivariable logistic regression analyses for the chronic subdural hematoma recurrence in 459 cerebral hemispheres with CSDHs.

	Univariable			Multivariable model 1			Multivariable model 2		
Factor	OR	95% CI	*p* value	OR	95% CI	*p* value	OR	95% CI	*p* value
Age (per 10-year increase)	0.85	0.61–1.19	0.36						
Male sex	2.21	0.95–5.14	0.07	1.64	0.68–3.94	0.27	1.69	0.70–4.04	0.24
Hypertension	0.66	0.33–1.32	0.24						
Diabetes mellitus	1.50	0.73–3.07	0.27						
Antiplatelet drugs	1.52	0.64–3.61	0.35						
Anticoagulant drugs	1.91	0.83–4.39	0.13						
Coagulopathy	1.66	0.60–4.55	0.33						
Eosinophil-rich	2.40	1.22–4.71	0.01^*^	2.63	1.31–5.27	0.007^*^	2.51	1.26–4.99	0.009^*^
Platelet count (per 5 × 10^3^/μL decrease)	1.36	1.05–1.76	0.02^*^	1.39	1.06–1.84	0.02^*^	−	−	−
Decreased platelet (<170 × 10^3^/μL)	2.17	1.09–4.30	0.03^*^	−	−	−	2.15	1.07–4.35	0.03^*^
Platelet transfusion	2.74	0.30–25.11	0.38						
Hematoma vol (10 mL per increase)	1.07	1.00–1.16	0.06	1.06	0.98–1.15	0.15	1.06	0.98–1.15	0.16
Contralateral surgery	0.82	0.33–2.03	0.67						

OR, Odds ratio; CI, Confidence interval. ^*^Indicates statistically significant (*p* < 0.05). Decreases in the PC are assessed as continuous and categorical variables in model-1 and -2, respectively.

### 3.4 Relationship between PC and eosinophil-rich blood

The PCs in the groups with (197 CSDHs) and without eosinophil-rich blood (280 CSDHs) were compared to evaluate the relationship between PCs and eosinophil-rich blood. No significant difference was observed between the groups (non-eosinophil-rich vs. eosinophil-rich: 211 ± 76 × 10^3^/μL vs. 217 ± 60 × 10^3^/μL; *p* = 0.38).

### 3.5 Thrombocytopenia and platelet transfusion

Regarding 16 surgery for 17 CSDHs with coexisting thrombocytopenia, platelet transfusions were performed before four surgeries for five CSDHs to suppress perioperative acute bleedings. In contrast, platelet transfusion was not administered for the remaining 12 surgeries for 12 CSDHs. PCs were significantly lower in the surgery with platelet transfusion than in that without transfusion (42 ± 18 × 10^3^/μL vs. 79 ± 13 × 10^3^/μL; *p* = 0.001).

In three of the four surgeries with platelet transfusion, PC did not increase over 100 × 10^3^/μL after platelet transfusion. In only one CSDH, PC increased over 100 × 10^3^/μL after the platelet transfusion. The effect of platelet transfusion was not sustained for a long time; on 5 or 6 days after surgery, the PC was not significantly different from those before the platelet transfusion (52 ± 22 × 10^3^/μL vs. 42 ± 18 × 10^3^/μL; *p* = 0.52).

One (20%) of the five CSDHs with platelet transfusion and 4 (33.3%) of the 12 CSDHs without platelet transfusion recurred, respectively. No significant differences were found between them (*p* > 0.99).

## 4 Discussion

Our study focused on the effect of PC values on postoperative recurrence of CSDH after burr hole surgery. Decrease in PC was shown to affect the postoperative recurrence of CSDH, and that risk was gradually increased in parallel with decrease in the PC. CSDH with preoperatively decreased PC (<170 × 10^3^/μL) recurred with double the frequency of those without a deceased PC (13.8 vs. 6.9%).

Continuous bleeding and exudation play pivotal roles in CSDH formation and progression. Ito et al. (11) reported a new hemorrhage accounting for 6.7% (range, 0.2–28.6%) of the hematoma content in 6–24 h. Bleeding is considered to be mediated by inflammation, angiogenesis, and fibrinolysis (5, 7, 12, 13). Numerous newly formed capillary vessels in the outer membrane of the CSDH tear easily and are permeable under focal inflammation, which can result in re-bleeding and exudation. Hemostatic disorders may facilitate CSDH progression and recurrence.

Platelets play a pivotal role in hemostasis; poor PC results in a bleeding tendency, and thrombocytopenia is associated with a high risk of intracranial hemorrhage (14). Therefore, a sufficient PC may be needed to prevent recurrence. In contrast, platelets have the potential to facilitate inflammation and may drive the regrowth of CSDH (13, 15). In patients with cancer, platelet transfusion was reported to increase perihematomal edema after intracerebral hemorrhage (15). Although platelets may have a two-sided effect on the postoperative recurrence of CSDH, the hemostatic effect of platelets may be more closely associated with the recurrence of CSDH, according to our findings. In the present study, antithrombotic therapy was not associated with the CSDH recurrence. Hemostatic disorder by decrease in PC may influence the CSDH stronger than inhibited hemostasis by antithrombotic agent. However, it may be attributed to mechanisms other than hemostatic disorder; in addition, deceased PC may reflect an unidentified factor. Further study is needed to elucidate the mechanisms.

A PC of 150–450 × 10^3^/μL is considered normal in adults (16). It can be affected by various conditions, including infection, cancer, leukemia, myelosuppression, liver cirrhosis, and malignancy (14–16). A PC > 100 × 10^3^/μL is believed to be needed for safe cranial surgery; even in emergency surgeries, a PC should be >80 × 10^3^/μL (8, 17). In the recurrence of CSDH, the effect of a decreased PC has not been well investigated and remains controversial (7, 18–21). The present study focused on PCs and CSDH recurrence and showed that CSDH recurrence was increased with a decrease in the PC. Moreover, even if a PC was within the normal range, CSDH with a greater PC recurred less frequently than CSDH with a lower PC. To facilitate the use of PCs in clinical settings, a PC value <170 × 10^3^/μL was defined as a “decreased PC” that might effectively predict recurrence. Threshold for decreased PC might be higher considering normal PC value (150–450 × 10^3^/μL) and other clinical conditions: in patients with cancer PC <60 × 10^3^/μL is associated with bleeding; in those with aplastic anemia, spontaneous fecal blood loss could occur at PC <10 × 10^3^/μL and remarkably increased at PC <5 × 10^3^/μL (22). However, bleeding risks because of a decrease in PC depend on the underlying disease. In anemic women with term singleton pregnancies, increased postpartum hemorrhage at PC <150 × 10^3^/μL was reported, where the threshold of PC was close to the one defined in the present study (23). During CSDH progression, continuous bleeding and exudation occur, which might be different from acute bleedings, resulting in higher threshold for decreased PC.

In an aging society, the use of antithrombotic agents increases (24). In addition to hemostasis disorder caused by a decreased PC, antithrombotic agents inhibit hemostasis and may theoretically be a risk factor for CSDH recurrence. In meta-analyses by Wang et al. and Poon et al., the use of antiplatelet and anticoagulant drugs increased the risk of recurrence (25, 26). In a meta-analysis review of randomized trials, Bakheet et al. (27) showed that the incidence of subdural hematoma was greater in patients using dual antiplatelet drugs than in those using aspirin alone. In contrast, in a retrospective study with a large sample size, Yu et al. (28) reported that antiplatelet therapy did not affect the recurrence rate of CSDH. In a systematic review by Nathan et al. (6), the use of anticoagulant medication was concluded to be associated with an increased re-bleeding risk with CSDH, but antiplatelet medication was not. In a multicenter, prospective cohort study with a large sample size, Poon et al. showed that neither antiplatelet nor anticoagulant drug use was associated with CSDH recurrence. In the present study, neither antiplatelet nor anticoagulant drug use was associated with CSDH recurrence. The effect of these drugs on CSDH recurrence remains controversial; it may be smaller than that of a decreased PC based on our findings. Further studies are needed to elucidate this issue.

The present study showed that eosinophil-rich blood was another independent risk factor for postoperative CSDH recurrence, as we have previously reported (7). In contrast, PCs were not associated with eosinophil-rich blood. A decreased PC and increased eosinophil count may relate to different mechanisms of CSDH recurrence, including hemostasis disorders and inflammation. We suggest that preoperative examination of platelet and eosinophil counts is important for predicting postoperative recurrence by assessing both hemostasis disorders and inflammation.

This study had some limitations. First, this study was retrospectively conducted at a single institution. Second, not all patients were included or evaluated. However, most patients were included in the analysis, and the selection bias might be small. Third, PCs were assessed at admission; however, changes were not evaluated during the postoperative course. However, the PC at admission might be affected by a patient’s general and homeostatic condition and effectively predict CSDH recurrence. Fourth, the effect of platelet transfusion on recurrence was not evaluated because of the small number of patients. However, the effect of platelet transfusion on the CSDH is considered limited because the increase in the PC from platelet transfusion is minimal and not sustained for a long period.

## 5 Conclusion

Our study showed that a decrease in the PC affected the postoperative recurrence of CSDH, and the risk was gradually increased in parallel with a decrease in the PC. CSDH with a preoperatively decreased PC (<170 × 10^3^/μL) recurred with double the frequency of those without a decreased PC. Therefore, CSDH patients with decreased PCs might require careful follow-up.

## Data availability statement

The raw data supporting the conclusions of this article will be made available by the authors, without undue reservation.

## Ethics statement

The studies involving humans were approved by Institutional review board of Kawasaki Medical School. The studies were conducted in accordance with the local legislation and institutional requirements. The ethics committee/institutional review board waived the requirement of written informed consent for participation from the participants or the participants’ legal guardians/next of kin.

## Author contributions

KY: Conceptualization, Data curation, Formal analysis, Investigation, Writing – original draft. MM: Data curation, Writing – review & editing. EK: Conceptualization, Writing – original draft. YM: Data curation, Writing – review & editing. TH: Supervision, Writing – review & editing.
