# Supplementary material for: Effect of decreased platelets on postoperative recurrence of chronic subdural hematoma
Source: Front Neurol. 2023 Dec 20;14:1308991. doi: 10.3389/fneur.2023.1308991 (PMC10765528; doi:10.3389/fneur.2023.1308991)
Supplement: Supplementary file 1 [file Data_Sheet_1.docx]

Supplementary Material

**Supplementary Figure S1**

488 CSDHs in 431 patients who underwent burr hole surgery between January 2013 and December 2022.

Four CSDHs in four patients were excluded because middle meningeal artery embolization was performed in combination with the burr hole surgery.

477 CSDHs in 421 patients met the inclusion criteria.

Seven CSDHs in six patients were excluded for not having preoperative platelet count data after the blood examination.

459 CSDHs in 405 patients were finally analyzed.

18 CSDHs in 16 patients were not followed-up.

**Supplementary Figure S2.**

Receiver operating characteristic curve of platelet decrease in chronic subdural hematoma recurrence


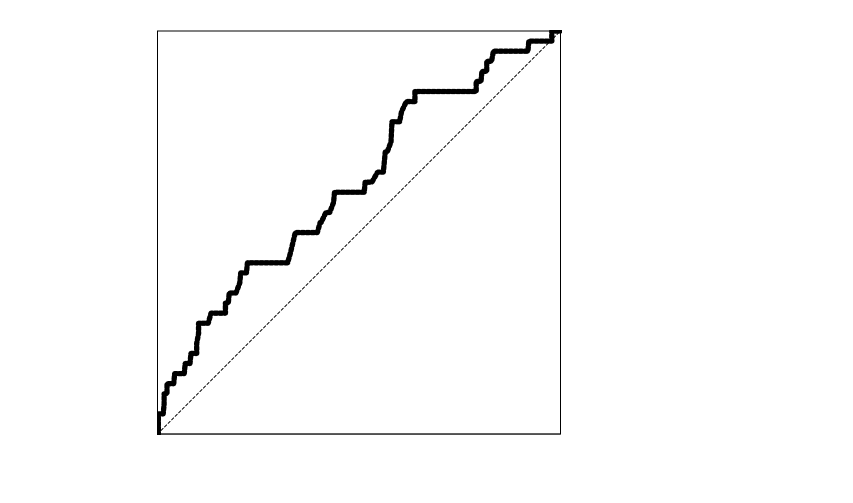


1

1

0

Sensitivity

1 − Specificity

0.385

0.224

Area under the curve, 0.60; 95% confidence interval, 0.51–0.69; *p* = 0.04.

Cut-off: 170×10^3^/μL provides 38.5% sensitivity and 77.6% specificity.
